# Efficacy and safety of zero‐fluoroscopy ablation of ventricular arrhythmias originating from the right ventricular outflow tract: Comparison with fluoroscopy‐guided ablation without a three‐dimensional electroanatomic mapping system

**DOI:** 10.1002/joa3.12815

**Published:** 2023-01-24

**Authors:** Ba Van Vu, Phong Dinh Phan, Linh Tran Pham, Kien Trung Hoang, Thinh Duc Do, Hung Manh Nguyen, Linh Thi Hai Ngo, Dung Tien Le, Nguyen Thao Phan, Huu Cong Nguyen, Thuc Cong Luong

**Affiliations:** ^1^ Cardiology Department Vietnam Military Medical University Hanoi Vietnam; ^2^ Cardiovascular Center E Hospital Hanoi Vietnam; ^3^ Cardiology Department Hanoi Medical University Hanoi Vietnam; ^4^ Vietnam National Heart Institute Bach Mai Hospital Hanoi Vietnam; ^5^ School of Clinical Medicine The University of Queensland Herston Queensland Australia; ^6^ University of Medicine and Pharmacy Hanoi National University Hanoi Vietnam; ^7^ Cardiology Department Military Hospital 103 Hanoi Vietnam

**Keywords:** right ventricular outflow tract, ventricular arrhythmias, zero‐fluoroscopy ablation

## Abstract

**Background:**

Radiofrequency catheter ablation is the preferred treatment choice for ventricular arrhythmias (VAs) originating from right ventricular outflow tract (RVOT) in symptomatic patients and is usually performed under fluoroscopy guidance. Zero‐fluoroscopy (ZF) ablations using 3D mapping system applied for treatment of various types of arrhythmias are trending and practiced in many centers around the world, but rarely done in Vietnam. The objective of this study was to evaluate the efficacy and safety of zero‐fluoroscopy ablation of RVOT VAs, compared with fluoroscopy‐guided ablation without a 3D electroanatomic mapping (EAM) system.

**Methods and Results:**

We conducted a nonrandomized, prospective single‐center study including 114 patients with RVOT VAs that had electrocardiographic features of typical left bundle branch block, inferior axis QRS morphology, and a precordial transition ≥ V_3_, from May 2020 to July 2022. The patients were assigned (without randomization) to two different approaches of either zero‐fluoroscopy ablation under the guidance of the Ensite system (ZF group) or fluoroscopy‐guided ablation without a 3D EAM (fluoroscopy group) in a 1:1 ratio. After a follow‐up time of 5.0 ± 4.9 months and 6.9 ± 9.3 months in the ZF and fluoroscopy groups, respectively, the results showed a higher success rate in the fluoroscopy group than in the complete ZF group (87.3% vs 86.8%), although the difference was not statistically significant. No major complication was noted in both the groups.

**Conclusion:**

ZF ablation for RVOT VAs can be done safely and effectively using the 3D electroanatomic mapping system. The results of ZF approach are comparable to that of the fluoroscopy‐guided approach without a 3D EAM system.

## 1 INTRODUCTION

Ventricular arrhythmias (VAs, including premature ventricular complexes [PVC], ventricular tachycardia [VT]) originating from the right ventricular outflow tract (RVOT) are common arrhythmias and often idiopathic.^1^, ^2^ Radiofrequency catheter ablation is the preferred treatment choice for RVOT VAs in symptomatic patients and is usually performed under fluoroscopy guidance.^3^ However, the use of fluoroscopy is associated with the risk of malignancies for patients and medical staff, as well as orthopedic injuries because of long‐term wearing of protective lead clothing/apron. Many practice guidelines as well as expert consensus have therefore advocated for an as low as possible radiation‐dose approach in electrophysiologic procedures to minimize the risk of ionizing radiation.^4^, ^5^ Zero‐fluoroscopy (ZF) ablations using 3D electroanatomical mapping (EAM) system for various types of arrhythmias has emerged as an effective alternative and is being performed in many centers worldwide,^6^, ^7^ but rarely done in Vietnam. Complete ZF approach for complicated arrhythmias such as atrial fibrillation and VAs originating from the left ventricle normally requires a combination of the 3D EAM system and intracardiac echocardiography (ICE), which ensures the safety of atrial septal puncture, as well as good catheter‐tissue contact.^8^, ^9^ However, ablation of less complex arrhythmias, such as VAs originated from right side of the heart, with the guidance of 3D EAM, can be performed with complete ZF without ICE assistance.^10^, ^11^ The results of recent studies have shown that ZF ablations under the guidance of a 3D EAM system for patients with idiopathic right‐sided VAs are safe and feasible.^9^, ^10^, ^11^, ^12^ Therefore, we aimed to evaluate, for the first time, the efficacy and safety of 3D‐guided zero‐fluoroscopy ablation for RVOT VAs, compared with fluoroscopy‐guided ablation without a 3D EAM system in a single center in Vietnam, where 3D ablations remain new and, in many cases, unaffordable.

## 2 MATERIALS AND METHODS

### 2.1 Study design

We conducted a nonrandomized prospective single‐center study including 114 patients with RVOT VAs from May 2020 to July 2022. The patients were assigned (without randomization) to two different approaches of either ZF or fluoroscopy group in a 1:1 ratio. The fluoroscopy approach used solely X‐ray imaging for the whole procedure without 3D EAM system. For ZF approach, we utilized an Ensite 3D EAM system (Abbott Laboratories Ltd.) without the use of X‐ray and medical staffs who assisted in these cases did not wear lead apron at the beginning of the procedure. The fluoroscopy system was used for backup of ZF procedures. Patients undergoing ablation with ZF approach that later required additional fluoroscopic support were excluded from the ZF group in the final analysis. This group of patients with minimal fluoroscopic (near‐zero fluoroscopy) approach was described separately. In this study, we focused on comparing procedural efficacy and safety in two groups: fluoroscopy‐guided approach without a 3D EAM system (or fluoroscopy group in short) and complete ZF approach with a 3D EAM system. Procedural efficacy was defined as the percentage of patients being free from VAs recurrence at the end of follow‐up. Primary safety endpoint was major complications, defined as death, cardiac tamponade, and permanent atrioventricular (AV) block. Secondary safety endpoint was minor complication, including vascular access complications and right bundle branch block. The study protocol was approved by the Ethics Committee of Hanoi E hospital and Vietnam Military Medical University.

### 2.2 Study population

All consecutive patients undergoing catheter ablation for RVOT VAs with electrocardiographic features of typical left bundle branch block (LBBB), inferior axis QRS morphology, and a precordial transition ≥ V_3_ were enrolled to this study. Prior to catheter ablation, the patients underwent laboratory screening such as echocardiography, electrocardiography (ECG), and 24‐h Holter monitoring to exclude those with structural heart disease and to determine VAs burden and morphology. Antiarrhythmic drugs were stopped for at least five half‐lives before the procedure. After a detailed discussion about two approaches regarding techniques, risks, and cost, a consensus about treatment option was made between the patient and the treating physician. All patients provided written informed consent to participate in this study.

### 2.3 Mapping and ablation protocol

Ensite Velocity 3D EAM system and Workmate electrophysiological system (St. Jude Medical Company) were used for catheter navigation, mapping, and activation mapping. Ablation catheters used were usually bidirectional deflectable with a tip size of 4–5 mm, either irrigated or nonirrigated.

#### 2.3.1 Zero‐fluoroscopy approach with a 3D EAM system

**FIGURE 1:**
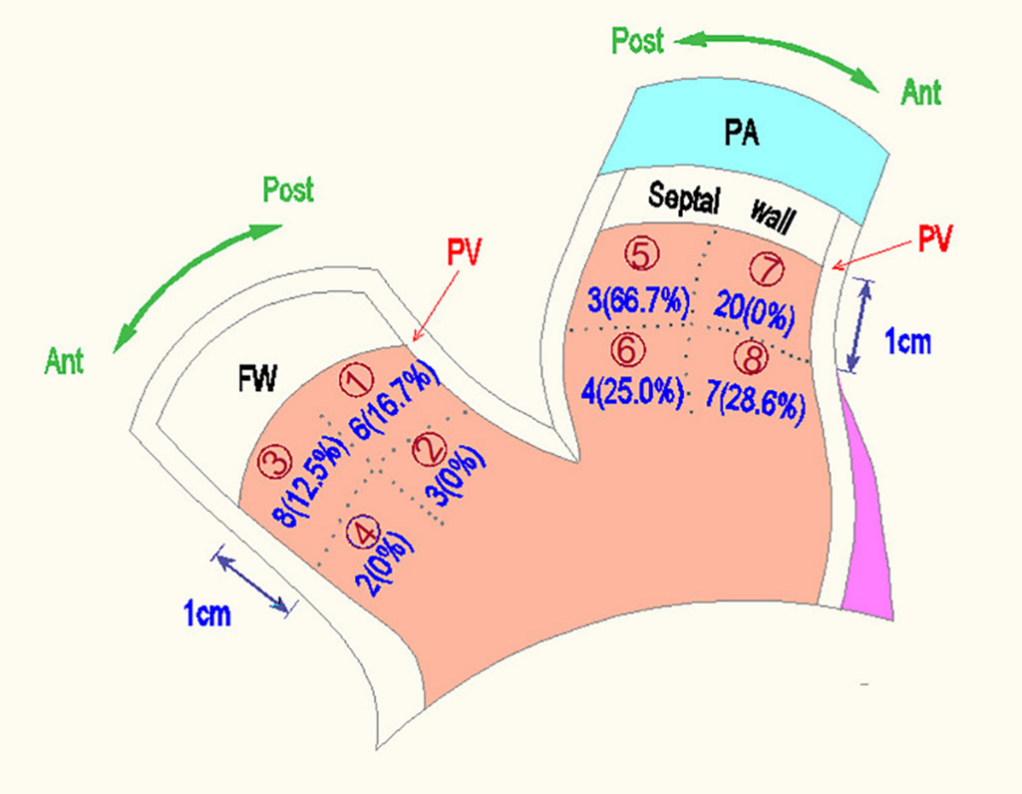
Representation of cases according to different anatomical locations in the RVOT and their recurrence rate. PA, pulmonary artery; FW, free‐wall; PV, pulmonary arterial valve; Ant, anterior; Post, posterior; *n* (%) = number of patients (recurrence rate). (1) free‐wall, posterior, and proximal side; (2) free‐wall, posterior, and distal side; (3) free‐wall, anterior, and proximal side; (4) free‐wall, anterior, and distal side; (5) septal wall, posterior, and proximal side; (6) septal wall, posterior, and distal side; (7) septal wall, anterior, and proximal side; and (8) septal wall, anterior, and distal side.

Catheters positioning and mapping were performed under the assistance of the Ensite Velocity 3D EAM system using nonsteerable diagnostic catheters (a decapolar catheter 5F, 2/8/2 mm and a quadripolar catheter 5F, 5/5/5 mm, St. Jude Medical Company). Fluoroscopy system was not used for this approach and set for backup. We used two geometric projections (left anterior oblique [LAO] and right anterior oblique [RAO]) to create a referential navigation for initial manipulation, then projections were modified according to operator's preference during mapping process. The external skin patch was used as the initial reference. The RVOT was anatomically divided into eight sites: (1) free‐wall, posterior, and proximal side, (2) free‐wall, posterior, and distal side, (3) free‐wall, anterior, and proximal side, (4) free‐wall, anterior, and distal side, (5) septal wall, posterior, and proximal side, (6) septal wall, posterior, and distal side, (7) septal wall, anterior, and proximal side, and (8) septal wall, anterior, and distal side. The region locating within 1 cm of the pulmonic valve was defined as the proximal side below the pulmonic valve, and the region locating at more than 1 cm away from the pulmonic valve was defined as the distal side below the pulmonic valve (Figure 1).^13^

##### Manipulation of the nonsteerable decapolar diagnostic catheter via the superior vena cava (SVC)

**FIGURE 2:**
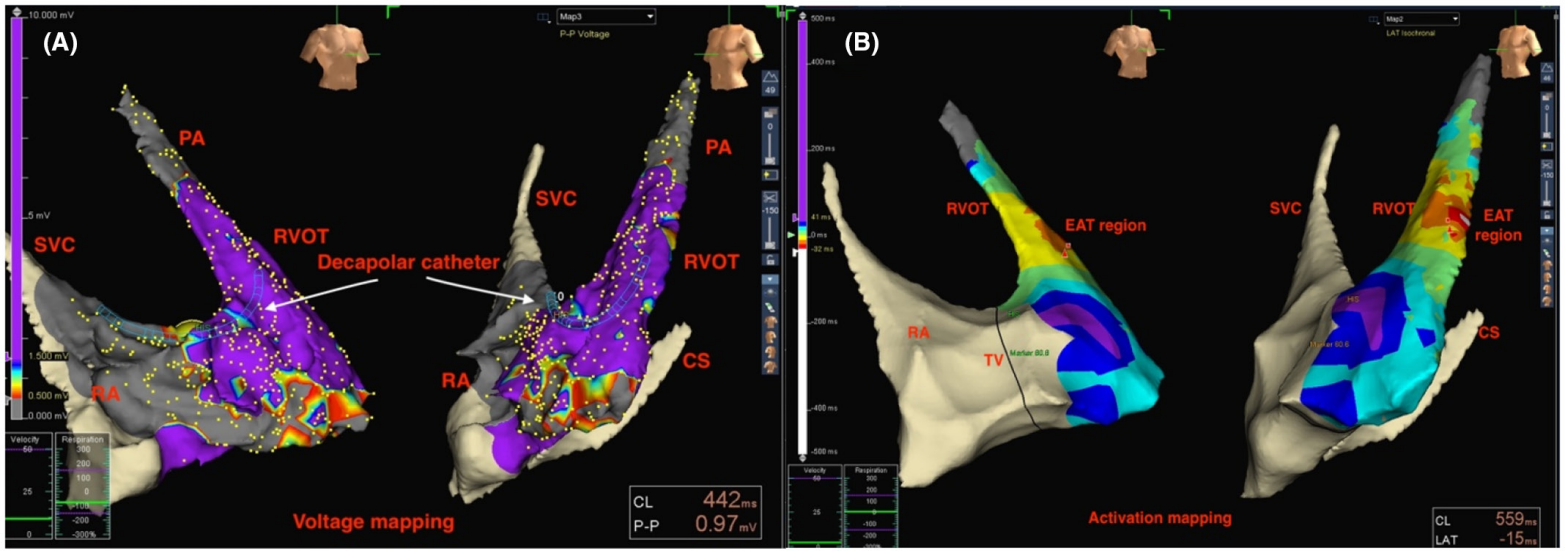
Utility of nonsteerable decapolar catheter for voltage mapping (A) and activation mapping (B) of RVOT Vas. PA, pulmonary artery; SVC, superior vena cava; RA, right atrium; TV, tricuspid valve; CS, coronary sinus; RVOT, right ventricular outflow tract; and EAT, earliest activation time.

The preshaped nonsteerable decapolar catheter was connected to the EAM system and advanced firstly into right atrium (RA) through the left subclavian or right jugular vein puncture, and then the SVC. The 3D geometry of SVC, RA, and RVOT were partly constructed by the advancement of the catheter as its position during movement was confirmed by the obtained intracardiac electrograms. When the catheter contacted the tricuspid annulus (TA), the His bundle signal was noted as a marker. We used the decapolar catheter to first build a voltage mapping to exclude underlying cardiomyopathy (Figure 2A), then to create activation mapping of RVOT VAs, and finally, color‐coded map (Figure 2B). After completing activation mapping, the catheter was pulled back and placed inside the coronary sinus (CS) and can then be set as an intracardiac reference for mapping confirmation by ablation catheter.

##### Manipulation of the nonsteerable quadripolar diagnostic catheter via the inferior vena cava (IVC)

The quadripolar catheter was used to reconstruct a 3D geometry roadmap of the IVC through the right femoral vein. The intracardiac electrograms were not presented until the catheter approached to RA. During advancement of catheter, it was kept forward superiorly and adjusted when stuck or misdirection was suspected.

##### Manipulation of the bidirectional ablation catheter

Based on the previously created 3D RVOT and IVC geometry by nonsteerable catheters, the ablation catheter, after being connected to RF generator, was advanced through the IVC, RA, and TA to RVOT following the 3D geometry roadmap. The bidirectional ablation catheter was used to further reconstruct and confirm the activation map that was previously done using the decapolar catheter. Pace mapping was subsequently done before RF application.

#### 2.3.2 Fluoroscopy‐guided approach without a 3D EAM system

Catheters used for mapping and ablation included one quadripolar catheter and one bidirectional ablation catheter. Fluoroscopy system was set up to use two geometric projections (LAO 60 degree and RAO 30 degree) for navigation. The advancement of catheters into RV structures (RV apex and RVOT) was performed via right femoral vein under fluoroscopic guidance only. The activation map was first implemented for identification of critical sites over roving the ablation catheter on RVOT areas from proximal to distal side. During mapping with ablation catheter, the sites of anterior/posterior sides and septal/free‐wall were visualized on RAO and LAO views, respectively. The origin of VAs foci determined by activation mapping would be verified by pace mapping. RF energy was applied at a site where earliest activation time and the most identical QRS morphology were noted.

### 2.4 Follow‐up

The patients were followed up after discharge at an outpatient clinic with 24‐h Holter monitoring. The recurrence status was identified as the presence of sustained VT, nonsustained VT, or greater than 1000 ventricular PVCs on 24‐h Holter ECG.^14^

### 2.5 Statistical analysis

Continuous variables are represented as the mean ± standard deviation (SD). Categorical data are described as percentages. Statistical Package for the Social Sciences (SPSS) version 13.0 (IBM Inc.) was used for data analysis. All analyses were two‐sided and a *p* < .05 was considered statistically different.

## 3 RESULTS

From May 2020 to July 2022, a total of 114 patients met inclusion criteria, all of whom were enrolled in this study and were divided into two groups: fluoroscopy approach (*n* = 55) and ZF approach (*n* = 59). In the ZF approach group, six patients required a conversion to supported fluoroscopy and were not included in the final analysis, resulting in 53 patients undergoing the complete ZF approach.

### 3.1 Baseline characteristics of patients

Table 1 represents the demographic and clinical characteristics, which shows no significant differences between two groups with all *p* > .05.

**TABLE 1:** Demographic and clinical characteristics of the study population

Baseline characteristics of patients	ZF (*n* = 53)	Fluoroscopy (*n* = 55)	*p* value
Age (years old)	52.6 ± 13.4	48.8 ± 14.1	.15
Female (%)	73.6%	80.0%	.43
Symptoms			
Chest pain (%)	60.4%	51.9%	.39
Dyspnea (%)	39.6%	40.4%	.93
Palpitation (%)	72.9%	76.9%	.64
Syncope/near‐syncope (%)	13.2%	20.4%	.32
Holter recordings			
PVC only (%)	77.4%	70.4%	.41
Nonsustained VT (%)	15.7%	26.4%	.18
Sustained VT (%)	0.0%	5.5%	.09
PVC/24 h	23849.3 ± 12551.7	22481.0 ± 10414.5	.55
Echo findings			
LVEF (%)	63.4 ± 10.2	64.7 ± 9.4	.48

Abbreviations: LVEF, left ventricular ejection fraction; PVC, premature ventricular complex; VT, ventricular tachycardia; ZF, zero‐fluoroscopy.

### 3.2 Procedural parameters and ablation outcomes

Procedural parameters and ablation outcomes were compared between two groups and summarized in Table 2. Average procedural time was comparable between two groups (67.9 vs 62.9 min in ZF and fluoroscopy groups, respectively) but the ZF approach has a shorter ablation time (503.4 vs 656.0 s). In the fluoroscopy group, the procedure was unsuccessful in one case, which we suspected because of an epicardial origin. Overall, the acute success rate of the two groups in the study was excellent, reaching 100% in the ZF group and 98.2% in the fluoroscopy group, but the difference was not statistically significant (*p* = .32). After a comparable follow‐up time (5.0 vs 6.9 months, *p* = .21), the results showed a numerically higher success rate in the fluoroscopy approach group than in the complete ZF group (87.3% vs 86.8%) although the difference did not reach statistical significance (*p* = .94). Regarding procedural safety, we had a few cases of transient and reversible right bundle branch block in both groups, but no major complications were observed (Table 2). Success rate was comparable between open irrigated and nonirrigated catheter in both ZF and fluoroscopy groups (*p* = .65 and *p* = .72, respectively). Among six patients requiring conversion to fluoroscopy approach, the success rate was low, at 66.7% (4/6 patients).

**TABLE 2:** Procedural parameters and ablation outcomes

Characteristics	ZF (*n* = 53)	Fluoroscopy (*n* = 55)	*p* value
Ablation catheter			
Open‐irrigated (*n*, %)	(27) 50.9%	(27) 49.1%	.85
Procedural results			
Total procedure time (min)	67.9 ± 22.2	62.9 ± 31.1	.34
Fluoroscopy time (s)	00.0 ± 00.0	718.5 ± 465.1	**<.001**
DAP (mGycm^2^/s)	00.0 ± 00.0	26493.6 ± 69499.1	**.007**
Total RF ablation time (s)	503.4 ± 263.5	656.0 ± 465.8	**.01**
Number of lesions	6.2 ± 3.6	8.7 ± 6.0	**.012**
Local EAT (ms)	28.8 ± 6.4	24.8 ± 4.4	**<.001**
Major complication (n, %)	0 (0.00%)	0 (0.00%)	‐
Follow‐up time (months)	5.0 ± 4.9	6.9 ± 9.3	.21
Acute success rate (%)	(53/53) 100%	(54/55) 98.2%	.32
Success rate (%)	(46/53) 86.8%	(48/55) 87.3%	.94
Open‐irrigated (%)	89.9%	89.9%	1.00
Nonirrigated (%)	84.6%	85.7%	.83

*Note*: The bold values indicate the statistical significance (*p* < .05).

Abbreviations: DAP, dose area product; EAT, earliest activation time; RF, radio frequency; ZF, zero‐fluoroscopy.

Figure 1 depicts the frequency of the original site of RVOT VAs foci. The most common site of origin was at the septal, anterior, and proximal side (the 7th site), accounting for 37.7% (20 cases). Moreover, ablation outcomes at this site were excellent, no recurrence was observed. Eliminating VAs foci originated at the second site (free‐wall, posterior, and distal side) and fourth site (free‐wall, left, and distal side) also had great outcomes with no recurrence, although the number of cases was small (three and two patients, respectively). The shortest procedure time (55.0 ± 8.66 min) was recorded at the second site, and the shortest RF time (382.5 ± 81.3 min) was delivered at the fourth site. In contrast, with VAs originating from the fifth site (septal, posterior, and proximal side), procedure and ablation time were relatively short (55 min and 435 s), but recurrence rate was the highest, at 66.7% (2/3 cases). Although the longest RF ablation time (567 s) was recorded when RF application was performed at the third site (free‐wall, left, and proximal side), procedural outcome was satisfactory with recurrence occurring in one out of eight cases (12.5%).

## 4 DISCUSSION

### 4.1 Safety and efficacy of zero‐fluoroscopy ablation for RVOT VAs compared with fluoroscopy approach

The main finding of our study is that the safety and efficacy of catheter ablation of RVOT VAs completely guided by EAM without the use of fluoroscopy were comparable to the fluoroscopy‐guided approach without a 3D EAM system.

The Ensite system used for ZF ablations was mentioned in several studies from 2007 to 2013.^11^, ^15^ Most of these studies were conducted with ablation of atrial flutter, atrial fibrillation, and paroxysmal supraventricular tachycardia, and only Von Bergen's study performed ablation completely without fluoroscopy in two patients with RVOT VAs with successful results and no recurrence at 1‐year follow‐up.^16^ Razminia's 5‐year experience of ZF ablations showed that such approach is effective and safe in a study published in 2017. This was the largest study in size with 500 patients with various types of arrhythmias. However, the VAs originated from RVOT were recorded in only 10 cases of PVC and one case of VT, and the results were not analyzed for this subgroup.^6^ Wang et al (2017) reported the use of Ensite NavX System without ICE for ZF ablation of idiopathic VAs compared with fluoroscopy approach in a multicenter study. Although the sub‐analysis on the efficacy and safety of the ZF approach was not noted in the subgroup undergoing ablation for RVOT VAs, the study demonstrated that using 3D EAM for completely ZF approach is as safe and efficient as the fluoroscopy approach for the ablation of idiopathic VAs.^11^ In a systematic review and meta‐analysis of ZF approach versus fluoroscopy approach for cardiac arrhythmia ablations published in 2021, the subgroup analysis of VAs ablation presented no significant differences in acute procedure success rate, recurrence‐free survival, periprocedural complication rate, or total procedural time between ZF and fluoroscopy approach.^17^

RVOT VAs are frequently idiopathic of origin.^1^, ^2^ However, some VAs originate from a underlying cause of cardiomyopathy, but present on electrocardiogram with a precordial transition ≥ V_3_, such as arrhythmogenic right ventricular cardiomyopathy (ARVC). We did not find any patients with cardiomyopathy under voltage maps in this study (Figure 2A). The use of the 3D EAM system in mapping VAs demonstrates the advantages of the ZF approach over the fluoroscopy approach. Among the initial ZF cases, we had six patients that needed additional fluoroscopy support because of the failure to terminate VPCs after a prolonged ablation and procedure time during complete ZF approach. The combination of fluoroscopy and 3D EAM system in these situations helped to rule out pseudo‐geometry, as well as ensure the procedural safety and efficacy. Moreover, reanalysis of ECG features of these six cases, we found the V2 transition ratio > 0.6 in three cases, so we converted to fluoroscopy approach to map other suspicious regions in the left ventricular (LV) outflow tract. These patients were excluded from the final analysis of our study.

The procedure time was comparable between two groups (*p* = .34), although extra time was needed in the ZF group to perform the voltage map, as well as the immature catheter manipulation in the first cases during VAs mapping. The difference of the ablation time and the number of lesions between groups supports the important role of the 3D EAM system compared with the alone fluoroscopy system. Because of the advantage of intuitive catheter visualization on the 3D map, it allowed more localized identification of the trigger area, as well as reduced the risk of catheter dislodgment and repeated ablation. Besides, the point‐by‐point construction of a 3D activation map was performed when a roving catheter has been stably contacted with the cardiac surface. We believe that the signal of bipolar electrodes local activation recorded during activation mapping of the ZF group may be more detailed and sharper than that of the fluoroscopy group. This could explain that the local earliest activation time measured in the ZF group was statistically longer than that of the fluoroscopy group in our study (*p* < .05).

The most common site of origin of RVOT VAs in our study was the anterior septal region and close to the pulmonary valve (proximal side), consistent with what reported by Kamakura et al (1998).^13^ VAs originating from the septal posterior proximal site and the septal anterior distal site had a lower incidence but a higher recurrence rate than those from other sites. We speculate that the low success rate may be because the VAs in these patients originated from the LVOT or LV summit. ECG patterns of VAs from these locations are quite similar because of adjacent anatomical relationship.

The utilization of irrigated tip catheters has been shown to improve ablation safety and efficacy for ventricular tachycardia because of the reduction of risk of thrombus, char formation, and the production of larger lesions.^18^, ^19^ Although the success rate was numerically better in open‐irrigated subgroup, but the difference was not statistically significant in this study.

The results of our study demonstrated that the efficacy and safety of a completely zero‐fluoroscopic approach of CA procedures for RVOT VAs were comparable to that of the fluoroscopy‐guided approach without a 3D EAM system. This study is consistent with the previous studies in terms of procedural time and total success rate. The EAM system‐based ZF approach not only minimizes the risk of ionizing radiation for healthcare staff and patients but also helps the medical staff avoid orthopedic injuries.

### 4.2 Utility of nonsteerable decapolar catheter for activation mapping of RVOT VAs

An ablation catheter could be used in isolation to perform activation mapping for zero‐fluoroscopy RVOT VAs ablation under the guidance of EAM system. In our opinion, the addition of diagnostic catheters to ablation catheter assisted a better localization and could be used as anatomical markers, enhancing the safety of the procedure. In this study, we used a nonsteerable decapolar diagnostic catheter in addition to the ablation catheter as an initial step for voltage and activation mapping of RVOT VAs (Figure 2). Using the nonsteerable decapolar catheter via SVC for mapping, RVOT VAs would assemble more points than using an ablation catheter only for the same period of time and has been demonstrated to be safe and feasible.^20^ Moreover, the nonsteerable catheter has the advantage of being easily preshaped and softer compared with deflectable catheters, which would avoid the risk of mechanical trauma. After creating the activation mapping by the nonsteerable decapolar catheter, an ablation catheter was advanced through IVC to access the RVOT to confirm activation map and 3D model.

## 5 LIMITATION

This is a single‐center study with small sample size, which limits the statistical power of any between‐group comparisons. Treatment assignment was done without randomization, which could introduce selection bias. In our study, because of patients' preference and economical affordability to EAM, we could not truly randomize our patients. The comparable characteristics of two groups, therefore, ensures the reliability of the study results. The inclusion criteria for the study were based solely on ECG features of typical LBBB, inferior axis QRS morphology, and a precordial transition ≥ V_3_, which may not reach complete ZF approach as the sites of foci outside of the RVOT cannot be excluded. It is necessary to analyze ECG features of VAs with other algorithms for localization to achieve procedure's ZF goal. Nevertheless, this study provides important preliminary outcomes data for an increasingly common approach worldwide but still considered new in Vietnam.

## 6 CONCLUSION

ZF ablation for RVOT VAs (VPC/VT) can be performed safely and effectively using the 3D EAM system. The results of ZF approach are comparable to that of the fluoroscopy‐guided approach without a 3D EAM system.

## CONFLICT OF INTEREST

No author has conflict of interest.

## FUNDING STATEMENT

None.

## PATIENT CONSENT

All patients provided written informed consent to participate in this study.

## ETHICS APPROVAL STATEMENT AND CLINICAL TRIAL REGISTRATION

The study protocol was approved by the Ethics Committee of Hanoi E hospital. This study was registered at Vietnam Military Medical University with Decision No. 2892/QĐ‐HVQY on July 7, 2020.
